# Relationship Between Blood Groups and Cardiovascular Diseases: Insights From an Algerian Inpatient Study

**DOI:** 10.7759/cureus.75624

**Published:** 2024-12-12

**Authors:** Selma Amrani, Khalil Chaouki Zemouri, Khelifa Abderrahmene Bouguerra, Yahia Cherifi, Rachid Bouhadad, Salim Benkhedda

**Affiliations:** 1 Population Genetics, University of Science and Technology Houari Boumediene (USTHB), Algiers, DZA; 2 Cardiology Oncology Collaborative Research Groupe, Faculty of Medicine, University of Algiers Benyoucef Benkhedda, Algiers, DZA; 3 Molecular Pathology, University Paul Sabatier Toulouse III, Toulouse, FRA

**Keywords:** abo blood-group system, cardiovascular diseases (cvd), hypertension, public health, rhesus group, risk factors cardiovascular diseases

## Abstract

Introduction: Research on the association between blood groups and cardiovascular diseases (CVDs) in Africa, including Algeria, is notably limited, with a primary focus on blood donors. This narrow scope hinders a comprehensive understanding of the genetic diversity of blood groups and their potential links to CVD risk within the African context. To bridge this knowledge gap, this study proposes to investigate the distribution of blood group genotypes and their association with CVD prevalence, aiming to enhance knowledge within the African context and contribute to global insights into the relationship between blood groups and CVD.

Methods: This retrospective study was conducted within the Cardiology A2 Department of Mustapha Bacha University Hospital (CHU) in Algiers, Algeria. The inclusion criteria comprised patients aged 18 years or older with confirmed diagnoses of CVDs. Conversely, patients without confirmed diagnoses or documented blood groups were excluded from the study. Data collection encompassed key cardiovascular risk factors, and blood groups were determined using standard serological testing methods. Statistical analyses were performed utilizing R and Jamovi software, with a predefined significance threshold of p < 0.05.

Results: Our analysis of a 2,780-patient cohort (61.7% male, mean age: 62.53 ± 13.06 years) revealed a predominance of blood type O (42.84%), followed by A (32.27%), B (16.19%), and AB (8.71%), with 93.42% of the cohort being Rhesus (Rh)-positive. The calculated ABO allele frequencies were 0.2315 (A), 0.1319 (B), and 0.6532 (O), indicating a significant deviation from Hardy-Weinberg equilibrium (HWE) (χ² = 47.88, p < 0.05), whereas the Rh distribution remained in equilibrium. Hypertension (94.50%), dyslipidemia (94.03%), and diabetes (79.57%) were the most prevalent cardiovascular risk factors. Acute coronary syndrome (ACS) was the leading diagnosis (39.75%), and notably, no significant associations were observed between blood groups and cardiovascular conditions or risk factors, except for a marginally higher prevalence of arrhythmia among Rh-negative (Rh−) individuals (9.29% vs. 5.81%).

Conclusion: This study provides novel insights into the distribution of ABO and Rh blood types among Algerian patients with CVD, highlighting a prevalence of Group O and Rh-positive (Rh+) individuals, though without significant associations between blood groups and major cardiovascular risk factors. Contrary to previous findings suggesting elevated cardiovascular risk in non-O blood groups, our results did not establish significant associations between blood groups and cardiovascular conditions or risk factors. The high prevalence of ACS, hypertension (particularly among older males), dyslipidemia, and smoking underscore the urgent need for targeted preventive strategies and further research to elucidate the complex interplay between blood types, genetic factors, and CVD in this population. These findings emphasize the necessity for addressing modifiable risk factors through education and preventive measures to improve cardiovascular outcomes.

## Introduction

Cardiovascular disease (CVD) remains the leading cause of premature mortality worldwide [1-3]. Despite significant advancements in prevention and risk assessment, CVD continues to present a substantial public health challenge [1]. The prevalence of CVD in Algeria is particularly concerning, accounting for nearly half of all deaths (46.2%), highlighting the critical need for comprehensive primary prevention strategies [4].

The ABO system classifies blood into four types, A, B, AB, and O, based on the presence of specific antigens on red blood cells. Blood group O is distinct because it lacks these antigens. Rhesus (Rh) status is determined by the presence or absence of Rh protein in red blood cells, resulting in Rh-negative (Rh−) or Rh-positive (Rh+) classifications [5].

Numerous studies have investigated the association between ABO blood types and various systemic diseases [6]. The relationship between blood groups and CVD is a complex and evolving field of research. Research indicates that individuals with blood types A, B, and AB have a higher risk of cardiovascular events, such as heart attacks and strokes, than those with type O blood [7,8]. For example, blood group A is strongly associated with an increased risk of venous thromboembolism, while blood group B is linked to a higher likelihood of developing type 2 diabetes, a known risk factor for CVD [7-9]. These findings have sparked interest in the underlying biological mechanisms, particularly the role of blood group antigens in inflammatory pathways and clotting factors.

However, this field has been marked by an ongoing scientific debate. While some studies have suggested meaningful links between blood group antigens and cardiovascular risk, others have reported no significant association [10]. This highlights the need for further investigation to resolve these discrepancies and enhance our understanding of the relationship between blood groups and CVDs.

In Africa, including Algeria, research on the relationship between blood groups and CVD is limited, with most studies focusing on blood donors rather than on broader population-based cohorts. This narrow focus restricts a comprehensive understanding of the genotypic and allelic diversity of blood groups and their potential links to CVD risk in African populations. To address this gap, we conducted this study to investigate the distribution of blood group genotypes and alleles and their association with CVD prevalence. By addressing this critical knowledge deficit, this study aimed to illuminate the genetic and clinical interplay of blood groups within the African context, thereby enriching global insights into these associations.

## Materials and methods

Study design

This retrospective study, based on medical records, was conducted in the Cardiology A2 Department of Mustapha Bacha University Hospital (CHU) in Algiers, Algeria. The study received ethical approval from the Ethics Committee of Mustapha Bacha University Hospital (authorization number: 0108, approved on January 15, 2024). Data collection spanned from February 1, 2024, to June 10, 2024.

The following criteria were applied:

Inclusion Criteria

Patients aged 18 years or older with a confirmed diagnosis of one or more of the following CVDs were included: myocarditis, acute coronary syndrome (ACS), peripheral arterial disease (PAD), congenital heart disease (CHD), aortic disease (AD), tachycardia, and arrhythmia. Patients with documented ABO and Rh blood groups were eligible for the study.

Exclusion Criteria

Patients were excluded if they lacked a confirmed diagnosis, comprehensive medical history, or documented ABO blood group. Other exclusion criteria included patients with tumors, leukemia (both affecting coagulation profiles), organ transplantation (due to immunosuppressive therapy effects), and duplicate records (for data integrity).

Data were collected on the presence or absence of key cardiovascular risk factors, including hypertension, diabetes, smoking, physical activity, obesity (assessed by BMI (weight/height^2^) ≥ 30), and dyslipidemia.

CVD diagnoses were established based on clinical evaluations such as echocardiography, electrocardiogram (ECG), and chest radiography. ABO blood groups were confirmed using standard serological methods.

Allelic frequency determination and Hardy-Weinberg equilibrium (HWE) analysis

For the ABO blood group system, allelic frequencies were calculated using Bernstein’s formula (1930), with adjustments for deviations (D) [11]. For the Rh system, frequencies were derived using the method of Landsteiner and Wiener (1949) [12]. HWE was assessed using the chi-square (χ^2^) test.

ABO Blood Group System

Bernstein’s formula estimates the frequencies of alleles $A$, $B$, and $O$ as follows:

\begin{equation} p=1-\sqrt{O+B},\quad q=1-\sqrt{O+A},\quad r=\sqrt{O}\nonumber \\ \end{equation}

Where $A$, $B$, and $O$ represent phenotype frequencies. Deviations ($D$) are corrected using the following equations:

\begin{equation} D=1-(p+q+r),\quad p^{\prime}=(1+D/2)p,\quad q^{\prime}=(1+D/2)q,\quad r^{\prime}=(1+D/2)(r+D/2)\nonumber \\ \end{equation}

Rh Blood Group System

Allele frequencies for $Rh^{+}$ and $Rh^{-}$ were calculated as follows:

\begin{equation} d=\sqrt{Rh},\quad D=1-\sqrt{Rh}\nonumber \\ \end{equation}

Where $Rh$ refers to the frequency of the $Rh^{-}$ phenotype.

HWE Testing

The chi-square test ($\chi^{2}$) was employed to verify HWE:

\begin{equation} \chi^{2}=\sum\frac{(\text{Observed Count}-\text{Expected Count})^{2}}{\text{Expected Count}}\nonumber \\ \end{equation}

Statistical analysis

Continuous variables were presented as mean ± SD, while categorical variables were reported as counts and proportions, frequencies, frequencies and percentages (n/N, %). Group comparisons for continuous variables were performed using the Wilcoxon rank-sum test (Mann-Whitney U test) for two groups and the Kruskal-Wallis rank-sum test for multiple groups. Categorical variables were analyzed using Pearson’s chi-squared test.

All statistical analyses were conducted using R (version 4.3) and Jamovi (version 2.5.4). A p-value of less than 0.05 was considered statistically significant.

## Results

Population characteristics

This comprehensive study analyzed baseline characteristics in a cohort of 2,780 patients, comprising 1,715 males (61.7%) and 1,065 females (38.3%), resulting in a sex ratio of 1.61. The mean age of the participants was 62.53 years (± 13.06), with males averaging 62.80 years and females 62.10 years, showing no significant difference between the sexes (p = 0.361). The distribution of age groups was consistent across genders, with 3.92% of participants aged 20 to 38 years, 28.56% aged 38 to 57 years, 52.59% aged 57 to 77 years, and 14.93% aged 77 to 95 years, indicating no significant variance based on sex (p = 0.911).

Blood type analysis revealed a predominant presence of group O (42.84%), followed by group A (32.27%), group B (16.19%), and group AB (8.71%), with no significant differences across genders (p = 0.369). The distribution of the Rh factor showed a high prevalence of Rh+ blood (93.42%), consistent across both sexes (p = 0.518).

Hypertension was identified as a critical health concern, affecting 94.50% of the overall population. Notably, all male participants had hypertension compared to 85.63% of females, highlighting a significant difference (p < 0.001). This condition’s prevalence increased with age, reflecting a concerning trend, as it rose from 86.24% in the youngest age group to 96.14% in the oldest (p < 0.001). Diabetes affected 79.57% of the cohort, showing no significant gender differences (p = 0.195), but it exhibited a dramatic rise with age, reaching 100% in the older age groups. The dyslipidemia prevalence showed a significant age-related increase (94.03% overall; p < 0.001), starting at 35.78% in the youngest group, rising to 87.91% in patients aged 38-57 years, and reaching 100% in both older groups (57-77 years and 77-95 years).

Obesity presented significant differences based on sex, with a higher prevalence among males (36.44%) compared to females (21.50%) (p < 0.001). The prevalence of obesity varied by age, peaking at 36.44% in males and significantly increasing in the oldest group of women, where it reached 65.78%. Smoking was reported exclusively among males, with a striking 100% prevalence in this group (p < 0.001), while no female participants indicated smoking status. The activity levels among participants were similar, with 28.53% engaging in regular physical activity, showing no significant differences between sexes (p = 0.653).

Menopause status, with 89.58% of female participants being postmenopausal, showed a clear age-related difference (p < 0.001), with no reports of menopause in the youngest age group (20 to 38 years) and a prevalence of 38.07% among participants aged 77 to 95 years.

In terms of cardiovascular conditions, acute coronary syndrome was the most prevalent diagnosis, affecting 39.75% of the participants, with similar rates between males (40.00%) and females (39.34%) (p = 0.833). Other conditions, including myocarditis, PAD, AD, CHD, tachycardia, and arrhythmia, showed no significant differences across gender or age groups (p > 0.05 for all conditions).

Detailed patient characteristics are presented in Tables 1 and 2, providing comprehensive documentation of the studied population’s attributes and clinical features.

**Table 1:** Baseline characteristics according to sex ^1^ Mean ± (SD); n / N (%); ^2^ Wilcoxon rank sum test; Pearson’s Chi-squared test. *p<0.05; **p<0.01; ***p<0.001. The following values represent chi-square (χ²) test statistics used to analyze the relationships between variables by sex: Age Class: χ²(3) = 0.535 ABO: χ²(3) = 3.154 Rh: χ²(1) = 0.417 Hypertension: χ²(1) = 260.73 Diabetes: χ²(1) = 1.682 Dyslipidemia: χ²(1) = 1.113 Obesity: χ²(1) = 68.912 Smoking: χ²(1) = 2780 Activity: χ²(1) = 0.202 Menopause: χ²(1) = 2338.874 Pathology: χ²(6) = 2.808 ACS: Acute coronary syndrome; AD: Aortic disease; CHD: Congenital heart disease; PAD: Peripheral arterial disease

Variable	Overall N = 2,780^1^	By Sex		p-value^2^
		Men N = 1,715^1^	Women N = 1,065^1^	
AGE	62.53 ± (13.06)	62.80 ± (12.90)	62.10 ± (13.31)	0.361
AGE CLASS				0.911
(20, 38)	109 / 2,780 (3.92%)	64 / 1,715 (3.73%)	45 / 1,065 (4.23%)	
(38, 57)	794 / 2,780 (28.56%)	487 / 1,715 (28.40%)	307 / 1,065 (28.83%)	
(57, 77)	1,462 / 2,780 (52.59%)	907 / 1,715 (52.89%)	555 / 1,065 (52.11%)	
(77, 95)	415 / 2,780 (14.93%)	257 / 1,715 (14.99%)	158 / 1,065 (14.84%)	
ABO				0.369
A	897 / 2,780 (32.27%)	561 / 1,715 (32.71%)	336 / 1,065 (31.55%)	
AB	242 / 2,780 (8.71%)	155 / 1,715 (9.04%)	87 / 1,065 (8.17%)	
B	450 / 2,780 (16.19%)	262 / 1,715 (15.28%)	188 / 1,065 (17.65%)	
O	1,191 / 2,780 (42.84%)	737 / 1,715 (42.97%)	454 / 1,065 (42.63%)	
Rh				0.518
Negative	183 / 2,780 (6.58%)	117 / 1,715 (6.82%)	66 / 1,065 (6.20%)	
Positive	2,597 / 2,780 (93.42%)	1,598 / 1,715 (93.18%)	999 / 1,065 (93.80%)	
Hypertension	2,627 / 2,780 (94.50%)	1,715 / 1,715 (100.00%)	912 / 1,065 (85.63%)	<0.001***
Diabetes	2,212 / 2,780 (79.57%)	1,378 / 1,715 (80.35%)	834 / 1,065 (78.31%)	0.195
Dyslipidemia	2,614 / 2,780 (94.03%)	1,619 / 1,715 (94.40%)	995 / 1,065 (93.43%)	0.292
Obesity	854 / 2,780 (30.72%)	625 / 1,715 (36.44%)	229 / 1,065 (21.50%)	<0.001***
Smoking	1,715 / 2,780 (61.69%)	1,715 / 1,715 (100.00%)	0 / 1,065 (0.00%)	<0.001***
Activity	793 / 2,780 (28.53%)	484 / 1,715 (28.22%)	309 / 1,065 (29.01%)	0.653
Menopause	954 / 2,780 (34.32%)	0 / 1,715 (0.00%)	954 / 1,065 (89.58%)	<0.001***
Pathology				0.833
ACS	1,105 / 2,780 (39.75%)	686 / 1,715 (40.00%)	419 / 1,065 (39.34%)	
AD	260 / 2,780 (9.35%)	166 / 1,715 (9.68%)	94 / 1,065 (8.83%)	
Arrhythmia	168 / 2,780 (6.04%)	106 / 1,715 (6.18%)	62 / 1,065 (5.82%)	
CHD	253 / 2,780 (9.10%)	155 / 1,715 (9.04%)	98 / 1,065 (9.20%)	
Myocarditis	399 / 2,780 (14.35%)	250 / 1,715 (14.58%)	149 / 1,065 (13.99%)	
PAD	353 / 2,780 (12.70%)	212 / 1,715 (12.36%)	141 / 1,065 (13.24%)	
Tachycardia	242 / 2,780 (8.71%)	140 / 1,715 (8.16%)	102 / 1,065 (9.58%)	

**Table 2:** Baseline characteristics according to age groups ^1^ Mean ± (SD); n / N (%); ^2^ Kruskal-Wallis rank sum test; Pearson’s Chi-squared test. *p<0.05; **p<0.01; ***p<0.001. The following values represent chi-square (χ²) test statistics used to analyze the relationships between variables by age groups: Sex: χ²(3) = 0.535 ABO: χ²(9) = 4.793 Rh: χ²(3) = 2.406 Hypertension: χ²(3) = 50.271 Diabetes: χ²(3) = 1508.079 Dyslipidemia: χ²(3) = 830.841 Obesity: χ²(3) = 593.913 Smoking: χ²(3) = 0.535 Activity: χ²(3) = 1405.350 Menopause: χ²(3) = 73.698 Pathology: χ²(18) = 14.081 AD: Aortic disease; CHD: Congenital heart disease; ACS: Acute coronary syndrome; PAD: Peripheral arterial disease

Variable	Overall N = 2,780^1^	By Age Groups				p-value^2^
		(20, 38) N = 109^1^	(38, 57) N = 794^1^	(57, 77) N = 1,462^1^	(77, 95) N = 415^1^	
AGE	62.53 ± (13.06)	32.55 ± (4.10)	49.81 ± (5.42)	66.10 ± (5.04)	82.16 ± (4.07)	<0.001***
Sex						0.911
Men	1,715 / 2,780 (61.69%)	64 / 109 (58.72%)	487 / 794 (61.34%)	907 / 1,462 (62.04%)	257 / 415 (61.93%)	
Women	1,065 / 2,780 (38.31%)	45 / 109 (41.28%)	307 / 794 (38.66%)	555 / 1,462 (37.96%)	158 / 415 (38.07%)	
ABO						0.852
A	897 / 2,780 (32.27%)	37 / 109 (33.94%)	264 / 794 (33.25%)	468 / 1,462 (32.01%)	128 / 415 (30.84%)	
AB	242 / 2,780 (8.71%)	7 / 109 (6.42%)	72 / 794 (9.07%)	119 / 1,462 (8.14%)	44 / 415 (10.60%)	
B	450 / 2,780 (16.19%)	20 / 109 (18.35%)	121 / 794 (15.24%)	240 / 1,462 (16.42%)	69 / 415 (16.63%)	
O	1,191 / 2,780 (42.84%)	45 / 109 (41.28%)	337 / 794 (42.44%)	635 / 1,462 (43.43%)	174 / 415 (41.93%)	
Rh						0.493
Negative	183 / 2,780 (6.58%)	11 / 109 (10.09%)	49 / 794 (6.17%)	96 / 1,462 (6.57%)	27 / 415 (6.51%)	
Positive	2,597 / 2,780 (93.42%)	98 / 109 (89.91%)	745 / 794 (93.83%)	1,366 / 1,462 (93.43%)	388 / 415 (93.49%)	
Hypertension	2,627 / 2,780 (94.50%)	94 / 109 (86.24%)	721 / 794 (90.81%)	1,413 / 1,462 (96.65%)	399 / 415 (96.14%)	<0.001***
Diabetes	2,212 / 2,780 (79.57%)	21 / 109 (19.27%)	314 / 794 (39.55%)	1,462 / 1,462 (100.00%)	415 / 415 (100.00%)	<0.001***
Dyslipidemia	2,614 / 2,780 (94.03%)	39 / 109 (35.78%)	698 / 794 (87.91%)	1,462 / 1,462 (100.00%)	415 / 415 (100.00%)	<0.001***
Obesity	854 / 2,780 (30.72%)	109 / 109 (100.00%)	193 / 794 (24.31%)	279 / 1,462 (19.08%)	273 / 415 (65.78%)	<0.001***
Smoking	1,715 / 2,780 (61.69%)	64 / 109 (58.72%)	487 / 794 (61.34%)	907 / 1,462 (62.04%)	257 / 415 (61.93%)	0.911
Activity	793 / 2,780 (28.53%)	0 / 109 (0.00%)	0 / 794 (0.00%)	378 / 1,462 (25.85%)	415 / 415 (100.00%)	<0.001***
Menopause	954 / 2,780 (34.32%)	0 / 109 (0.00%)	241 / 794 (30.35%)	555 / 1,462 (37.96%)	158 / 415 (38.07%)	<0.001***
Pathology						0.724
ACS	1,105 / 2,780 (39.75%)	40 / 109 (36.70%)	304 / 794 (38.29%)	596 / 1,462 (40.77%)	165 / 415 (39.76%)	
AD	260 / 2,780 (9.35%)	7 / 109 (6.42%)	66 / 794 (8.31%)	141 / 1,462 (9.64%)	46 / 415 (11.08%)	
Arrhythmia	168 / 2,780 (6.04%)	7 / 109 (6.42%)	46 / 794 (5.79%)	88 / 1,462 (6.02%)	27 / 415 (6.51%)	
CHD	253 / 2,780 (9.10%)	12 / 109 (11.01%)	76 / 794 (9.57%)	128 / 1,462 (8.76%)	37 / 415 (8.92%)	
Myocarditis	399 / 2,780 (14.35%)	15 / 109 (13.76%)	123 / 794 (15.49%)	205 / 1,462 (14.02%)	56 / 415 (13.49%)	
PAD	353 / 2,780 (12.70%)	19 / 109 (17.43%)	102 / 794 (12.85%)	190 / 1,462 (13.00%)	42 / 415 (10.12%)	
Tachycardia	242 / 2,780 (8.71%)	9 / 109 (8.26%)	77 / 794 (9.70%)	114 / 1,462 (7.80%)	42 / 415 (10.12%)	

Analysis of genetic frequencies of ABO and Rh blood groups

Phenotypic Frequencies

**Figure 1:**
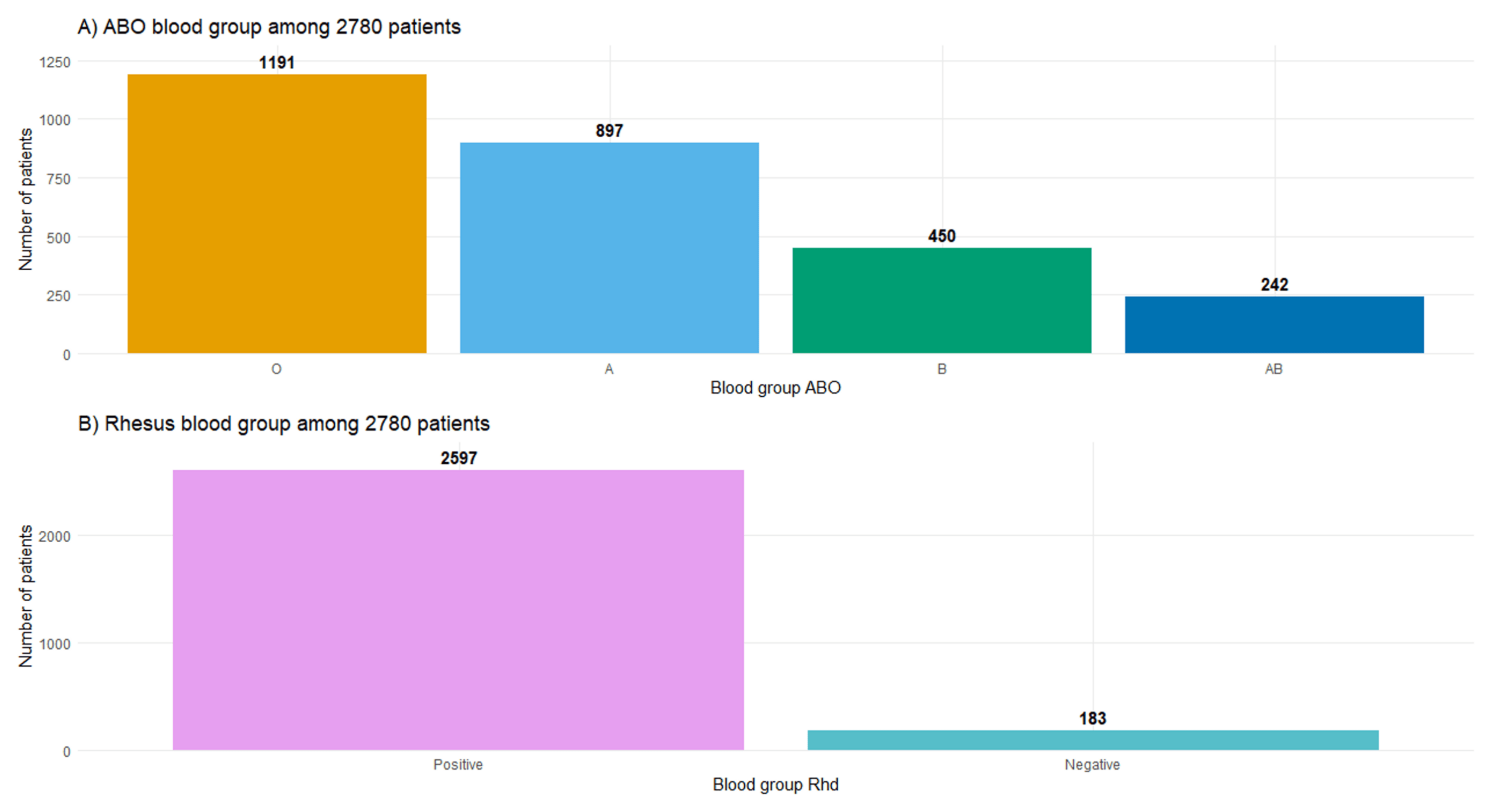
Blood groups phenotypes distribution

In a cohort of 2,780 patients, the phenotypic distribution of ABO blood groups (Figure 1, Panel A) was as follows: group A accounted for 32.27% (n = 897), group O for 42.84% (n = 1,191), group B for 16.19% (n = 450), and group AB for 8.71% (n = 242). Additionally, analysis of Rh blood group phenotypes (Figure 1, Panel B) in the same population revealed a predominance of Rh+ individuals, comprising 93.42% (n = 2,597), while Rh− individuals represented 6.58% (n = 183).

Allele Frequencies

The adjusted allele frequencies for the ABO blood group system, calculated using Bernstein's formula with D-deviation correction, were as follows: allele A (p') at 0.2315, allele B (q') at 0.1319, and allele O (r') at 0.6532 (Table 3). For the Rh blood group, allelic frequencies determined using the Landsteiner and Wiener method were 0.7435 for Rh+ and 0.2565 for Rh−.

**Table 3:** Calculation of allele frequencies for ABO blood groups

Allele	Frequency (Raw)	Correction (D)	Adjusted Frequency
A (p)	0.2317	-0.0016	0.2315
B (q)	0.1334	-0.0016	0.1318
O (r)	0.6545	-0.0016	0.6532

Genotypic Frequencies

The calculation of genotype frequencies for ABO blood groups, assuming HWE, yields the results shown in Table 4.

**Table 4:** Genotype frequencies for ABO blood groups under HWE HWE: Hardy-Weinberg equilibrium

Genotype	Frequency	Calculation
AA	0.0526	p² = (0.2315)²
BB	0.0174	q² = (0.1318)²
OO	0.4076	r² = (0.6532)²
AB	0.0606	2pq = 2 × 0.2315 × 0.1318
AO	0.2929	2pr = 2 × 0.2315 × 0.6532
BO	0.1685	2qr = 2 × 0.1318 × 0.6532

For the Rh blood group, researchers calculate the genotype frequencies, also assuming HWE, as shown in Table 5.

**Table 5:** Genotype frequencies for Rh blood groups under HWE Rh: Rhesus; HWE: Hardy-Weinberg equilibrium

Genotype	Frequency	Calculation
DD	0.5528	p² = (0.7435)²
Dd	0.3814	2pq = 2 × 0.7435 × 0.2565
dd	0.0658	q² = (0.2565)²

HWE 

The expected phenotypic frequencies for the ABO blood groups, calculated under HWE, were as follows: group A at 34.55% (0.3455), group B at 18.59% (0.1859), group AB at 6.06% (0.0606), and group O at 40.76% (0.4076). For a total sample size of 2,780 individuals, the theoretical or expected counts for each phenotype are detailed in Table 6.

**Table 6:** Expected ABO phenotypes under HWE HWE: Hardy-Weinberg equilibrium

Phenotype	Expected Frequency	Expected Count	Calculation
A	0.34596	49	(p² + 2pr) × N
B	0.185516	16.81	(q² + 2qr) × N
AB	0.060168	8.472	2pq × N
O	0.407113	33.13	r² × N

The expected phenotypic frequencies for the ABO blood groups, calculated under HWE, were compared to the observed frequencies. A chi-square analysis indicated that the distribution of ABO blood groups deviates significantly from HWE (Χ² = 47.88, degrees of freedom = 3, critical Χ² value (at p = 0.05) = 7.82). In contrast, the observed and expected frequencies for the Rh blood group were consistent with HWE.

CVDs and blood groups

The patients' characteristics, stratified by ABO and Rh blood groups (Tables 7, 8), showed no significant differences in age distribution among different ABO blood groups (p = 0.558) or between Rh− and Rh+ individuals (p = 0.340 and p = 0.493, respectively). The sex distribution also remained consistent across both ABO and Rh groups, with males constituting approximately 61.69% of the overall population and no significant differences noted among the groups (p = 0.369 for ABO and p = 0.518 for Rh).

**Table 7:** Patients characteristics according to ABO blood groups ^1^ Mean ± (SD); n / N (%); ^2^ Kruskal-Wallis rank sum test; Pearson’s Chi-squared test. *p<0.05; ** p<0.01; *** p<0.001. The following values represent chi-square (χ²) test statistics used to analyze the relationships between variables by ABO blood groups: Age Class: χ²(3) = 0.535 ABO: χ²(3) = 3.154 Rh: χ²(1) = 17.543 Hypertension: χ²(3) = 3.492 Diabetes: χ²(3) = 2.547 Dyslipidemia: χ²(3) = 1.001 Obesity: χ²(3) = 1.440 Smoking: χ²(3) = 3.154 Activity: χ²(3) = 2.582 Menopause: χ²(3) = 4.124 Pathology: χ²(18) = 24.551 ACS: Acute coronary syndrome; AD: Aortic disease; CHD: Congenital heart disease; PAD: Peripheral arterial disease

Variable	Overall N = 2,780^1^	By ABO Type				p-value^2^
		A N = 897^1^	AB N = 242^1^	B N = 450^1^	O N = 1,191^1^	
AGE	62.53 ± (13.06)	62.08 ± (12.99)	63.15 ± (12.79)	62.91 ± (13.12)	62.60 ± (13.15)	0.558
AGE CLASS						0.852
(20, 38)	109 / 2,780 (3.92%)	37 / 897 (4.12%)	7 / 242 (2.89%)	20 / 450 (4.44%)	45 / 1,191 (3.78%)	
(38, 57)	794 / 2,780 (28.56%)	264 / 897 (29.43%)	72 / 242 (29.75%)	121 / 450 (26.89%)	337 / 1,191 (28.30%)	
(57, 77)	1,462 / 2,780 (52.59%)	468 / 897 (52.17%)	119 / 242 (49.17%)	240 / 450 (53.33%)	635 / 1,191 (53.32%)	
(77, 95)	415 / 2,780 (14.93%)	128 / 897 (14.27%)	44 / 242 (18.18%)	69 / 450 (15.33%)	174 / 1,191 (14.61%)	
Sex						0.369
Men	1,715 / 2,780 (61.69%)	561 / 897 (62.54%)	155 / 242 (64.05%)	262 / 450 (58.22%)	737 / 1,191 (61.88%)	
Women	1,065 / 2,780 (38.31%)	336 / 897 (37.46%)	87 / 242 (35.95%)	188 / 450 (41.78%)	454 / 1,191 (38.12%)	
Rh						<0.001***
Negative	183 / 2,780 (6.58%)	52 / 897 (5.80%)	18 / 242 (7.44%)	13 / 450 (2.89%)	100 / 1,191 (8.40%)	
Positive	2,597 / 2,780 (93.42%)	845 / 897 (94.20%)	224 / 242 (92.56%)	437 / 450 (97.11%)	1,091 / 1,191 (91.60%)	
Hypertension	2,627 / 2,780 (94.50%)	856 / 897 (95.43%)	230 / 242 (95.04%)	419 / 450 (93.11%)	1,122 / 1,191 (94.21%)	0.322
Diabetes	2,212 / 2,780 (79.57%)	706 / 897 (78.71%)	201 / 242 (83.06%)	362 / 450 (80.44%)	943 / 1,191 (79.18%)	0.467
Dyslipidemia	2,614 / 2,780 (94.03%)	840 / 897 (93.65%)	230 / 242 (95.04%)	421 / 450 (93.56%)	1,123 / 1,191 (94.29%)	0.801
Obesity	854 / 2,780 (30.72%)	267 / 897 (29.77%)	71 / 242 (29.34%)	147 / 450 (32.67%)	369 / 1,191 (30.98%)	0.696
Smoking	1,715 / 2,780 (61.69%)	561 / 897 (62.54%)	155 / 242 (64.05%)	262 / 450 (58.22%)	737 / 1,191 (61.88%)	0.369
Activity	793 / 2,780 (28.53%)	238 / 897 (26.53%)	71 / 242 (29.34%)	133 / 450 (29.56%)	351 / 1,191 (29.47%)	0.461
Menopause	954 / 2,780 (34.32%)	299 / 897 (33.33%)	80 / 242 (33.06%)	173 / 450 (38.44%)	402 / 1,191 (33.75%)	0.248
Pathology						0.138
ACS	1,105 / 2,780 (39.75%)	355 / 897 (39.58%)	93 / 242 (38.43%)	185 / 450 (41.11%)	472 / 1,191 (39.63%)	
AD	260 / 2,780 (9.35%)	96 / 897 (10.70%)	26 / 242 (10.74%)	34 / 450 (7.56%)	104 / 1,191 (8.73%)	
Arrhythmia	168 / 2,780 (6.04%)	54 / 897 (6.02%)	17 / 242 (7.02%)	32 / 450 (7.11%)	65 / 1,191 (5.46%)	
CHD	253 / 2,780 (9.10%)	80 / 897 (8.92%)	14 / 242 (5.79%)	51 / 450 (11.33%)	108 / 1,191 (9.07%)	
Myocarditis	399 / 2,780 (14.35%)	114 / 897 (12.71%)	28 / 242 (11.57%)	70 / 450 (15.56%)	187 / 1,191 (15.70%)	
PAD	353 / 2,780 (12.70%)	112 / 897 (12.49%)	39 / 242 (16.12%)	46 / 450 (10.22%)	156 / 1,191 (13.10%)	
Tachycardia	242 / 2,780 (8.71%)	86 / 897 (9.59%)	25 / 242 (10.33%)	32 / 450 (7.11%)	99 / 1,191 (8.31%)	

**Table 8:** Patients characteristics according to Rh blood groups ^1^ Mean ± (SD); n / N (%); ^2^ Wilcoxon rank sum test; Pearson’s Chi-squared test. *p<0.05; **p<0.01; ***p<0.001. The following values represent chi-square (χ²) test statistics used to analyze the relationships between variables by Rh blood groups: Age Class: χ²(3) = 2.406 Sex: χ²(1) = 0.417 ABO: χ²(3) = 17.543 Hypertension: χ²(1) = 2.893 Diabetes: χ²(1) = 0.469 Dyslipidemia: χ²(1) = 3.842 Obesity: χ²(1) = 0.213 Smoking: χ²(1) = 0.417 Activity: χ²(1) = 0.294 Menopause: χ²(1) = 0.598 Pathology: χ²(6) = 4.621 Rh: Rhesus; ACS: Acute coronary syndrome; AD: Aortic disease; CHD: Congenital heart disease; PAD: Peripheral arterial disease

Variable	Overall N = 2,780^1^	By Rh Type		p-value^2^
		Negative N = 183^1^	Positive N = 2,597^1^	
AGE	62.53 ± (13.06)	61.57 ± (14.19)	62.60 ± (12.98)	0.340
AGE CLASS				0.493
(20, 38)	109 / 2,780 (3.92%)	11 / 183 (6.01%)	98 / 2,597 (3.77%)	
(38, 57)	794 / 2,780 (28.56%)	49 / 183 (26.78%)	745 / 2,597 (28.69%)	
(57, 77)	1,462 / 2,780 (52.59%)	96 / 183 (52.46%)	1,366 / 2,597 (52.60%)	
(77, 95)	415 / 2,780 (14.93%)	27 / 183 (14.75%)	388 / 2,597 (14.94%)	
Sex				0.518
Men	1,715 / 2,780 (61.69%)	117 / 183 (63.93%)	1,598 / 2,597 (61.53%)	
Women	1,065 / 2,780 (38.31%)	66 / 183 (36.07%)	999 / 2,597 (38.47%)	
ABO				<0.001***
A	897 / 2,780 (32.27%)	52 / 183 (28.42%)	845 / 2,597 (32.54%)	
AB	242 / 2,780 (8.71%)	18 / 183 (9.84%)	224 / 2,597 (8.63%)	
B	450 / 2,780 (16.19%)	13 / 183 (7.10%)	437 / 2,597 (16.83%)	
O	1,191 / 2,780 (42.84%)	100 / 183 (54.64%)	1,091 / 2,597 (42.01%)	
Hypertension	2,627 / 2,780 (94.50%)	178 / 183 (97.27%)	2,449 / 2,597 (94.30%)	0.089
Diabetes	2,212 / 2,780 (79.57%)	142 / 183 (77.60%)	2,070 / 2,597 (79.71%)	0.493
Dyslipidemia	2,614 / 2,780 (94.03%)	166 / 183 (90.71%)	2,448 / 2,597 (94.26%)	0.050*
Obesity	854 / 2,780 (30.72%)	59 / 183 (32.24%)	795 / 2,597 (30.61%)	0.644
Smoking	1,715 / 2,780 (61.69%)	117 / 183 (63.93%)	1,598 / 2,597 (61.53%)	0.518
Activity	793 / 2,780 (28.53%)	49 / 183 (26.78%)	744 / 2,597 (28.65%)	0.588
Menopause	954 / 2,780 (34.32%)	58 / 183 (31.69%)	896 / 2,597 (34.50%)	0.439
Pathology				0.593
ACS	1,105 / 2,780 (39.75%)	72 / 183 (39.34%)	1,033 / 2,597 (39.78%)	
AD	260 / 2,780 (9.35%)	15 / 183 (8.20%)	245 / 2,597 (9.43%)	
Arrhythmia	168 / 2,780 (6.04%)	17 / 183 (9.29%)	151 / 2,597 (5.81%)	
CHD	253 / 2,780 (9.10%)	19 / 183 (10.38%)	234 / 2,597 (9.01%)	
Myocarditis	399 / 2,780 (14.35%)	23 / 183 (12.57%)	376 / 2,597 (14.48%)	
PAD	353 / 2,780 (12.70%)	22 / 183 (12.02%)	331 / 2,597 (12.75%)	
Tachycardia	242 / 2,780 (8.71%)	15 / 183 (8.20%)	227 / 2,597 (8.74%)	

The prevalence of Rh positivity was significantly higher compared to Rh negativity (93.42% vs. 6.58%, p < 0.001). Stratification by ABO blood groups revealed that the O blood group was the most common (42.84%), followed by A (32.27%), B (16.19%), and AB (8.71%). Among Rh− individuals, group O was predominant (54.64%), whereas Rh+ individuals had distributions closely resembling the overall cohort (p < 0.001).

No significant differences were found in the prevalence of hypertension, diabetes, dyslipidemia, obesity, smoking, or physical activity levels across ABO or Rh blood groups. Hypertension was present in 94.50% of the overall cohort, with similar rates across all ABO types (p = 0.322) and slightly higher prevalence in Rh− individuals (97.27% vs. 94.30%, p = 0.089). Dyslipidemia was also highly prevalent (94.03%) and showed no significant variation by ABO type (p = 0.801), but Rh− individuals exhibited a slightly lower prevalence than Rh+ individuals (90.71% vs. 94.26%, p = 0.050). Obesity affected 30.72% of the population, with no notable differences across ABO (p = 0.696) or Rh (p = 0.644) groups. Smoking and physical activity rates similarly showed no significant associations with either blood group system (p ≥ 0.369).

The distribution of underlying pathologies was also analyzed. Acute coronary syndrome was the most frequent pathology, affecting 39.75% of the cohort, with no significant differences across ABO (p = 0.138) or Rh groups (p = 0.593). AD, arrhythmia, CHD, myocarditis, PAD, and tachycardia showed no significant associations with either blood group system. However, arrhythmia was slightly more common in Rh− individuals (9.29% vs. 5.81%).

## Discussion

In this retrospective study, we analyzed the distribution of ABO and Rh blood types among 2,780 hospitalized patients in Algeria, exploring their associations with CVD and its risk factors. Our findings revealed a predominance of blood group O and Rh+ individuals; however, we found no significant associations between blood groups and cardiovascular risk factors, suggesting that lifestyle and genetic factors may have a more decisive influence on CVD development in our population. Notably, our results differ from prior literature that suggested various associations between blood types and CVD, allowing us to better contextualize our findings within the existing body of knowledge.

The demographic characteristics of our study population mirror those often seen in cardiovascular research, with a majority of male participants (61.7%, N =175), reflecting the higher burden of CVD among men [1,2,13]. The similar, however non-significant, mean ages of men and women (62.80 vs. 62.10 years) suggest that cardiovascular issues appear at comparable ages across genders in this population.

ABO blood group distributions vary by region worldwide. In our cohort, the distribution of ABO blood groups revealed that Group O was the most common (42.84%), followed by Groups A (32.27%), B (16.19%), and AB (8.71%). This distribution, O>A>B>AB, as in donor's Algerian studies, differs from some African studies that found A>O>B>AB and from European and Indian formulas [13-15]. There was no significant difference in the distribution of the ABO groups between men and women (p = 0.369). Additionally, the Rh blood group was predominantly positive (93.42%) in the overall population, with a 6.58% prevalence of Rh− blood, as in previous Algerian donor studies [14]. No significant gender differences were observed in Rh positivity (p = 0.518).

Hypertension affected 94.50% of our study population, with all male participants and 85.63% of females affected, indicating a significant gender difference (p < 0.001) consistent with the study by Fuchs and Whelton [16]. Prevalence increased with age, from 86.24% in the youngest group to 96.14% in the oldest (p < 0.001), reflecting the established link between aging and cardiovascular risk. No significant differences in hypertension prevalence were observed across ABO types (p = 0.322), though rates were slightly higher in Rh− individuals (97.27% vs. 94.30%, p = 0.089), aligning with [17]. These findings suggest that lifestyle and genetic predispositions, rather than blood groups, may play a more significant role in hypertension development, warranting further research.

Our cohort exhibited a diabetes prevalence of 79.57%, with no significant gender differences (p = 0.195). However, diabetes prevalence increased dramatically with age, reaching 100% in older age groups (aged group of 57 to 77 years) and aged group of 77 to 95years, p < 0.001), while the youngest group (aged group of 20 to 38 years) had a prevalence of 19.27%. Importantly, there were no significant differences in diabetes prevalence across ABO blood groups (p = 0.467). These findings align with Ghafar et al. and Alqahtani et al. but contrast with Parente et al., who linked blood group B to ischemic heart disease in type 1 diabetes, and Getawa et al., who associated it with a higher risk of type 2 diabetes and blood group O with a lower risk [18-21].

Dyslipidemia was highly prevalent in our study (94.03%), with rates increasing from 35.78% in the youngest age group to 100% in the oldest (p < 0.001). No significant differences were found across ABO or Rh blood groups, consistent with the study by Neshat et al., which reported weak associations between blood groups and CVD risk factors [22]. Our results showed no significant variation by ABO type (p = 0.801), but Rh− individuals had a slightly lower prevalence than Rh+ individuals (90.71% vs. 94.26%, p = 0.050), contrasting with Shaikh et al., who found associations between ABO blood groups and lipid profiles in younger populations [23]. These findings mirror those in diverse populations [24]. While dyslipidemia is widespread, further research is needed to explore the impact of blood group types on lipid profiles and cardiovascular risk, with regular monitoring and targeted interventions crucial for mitigating its burden.

Our study found higher obesity prevalence among males (36.44%) than females (21.50%, p < 0.001), consistent with a North African study reporting similar trends in Algeria and Tunisia [25]. Obesity peaked at 65.78% in older women, with an overall prevalence of 30.72%. No significant differences were observed across ABO (p = 0.696) or Rh (p = 0.644) blood groups, contrasting with findings from Brazil but aligning with a Saudi study showing no association between blood groups and obesity in diabetics [19,26]. These results highlight the need for further investigation into obesity determinants across populations.

Our study reveals a concerning 100% smoking prevalence among male participants, with no female smokers reported (p < 0.001). This gender disparity aligns with findings in the study by Gallucci et al., which highlights women’s increased vulnerability to CVDs compared to men with similar smoking exposure [27]. In terms of physical activity, 28.53% of participants engaged in regular exercise, with no significant differences between sexes (p = 0.653), consistent with guidelines from the American Heart Association (AHA) and World Health Organization (WHO).

We found no significant associations between smoking or physical activity levels and ABO or Rh blood groups (p ≥ 0.369), contrasting with the study by MacDonald et al., which linked smoking to increased dyslipidemia risk [28]. These findings underscore the importance of addressing smoking and promoting physical activity in both sexes to mitigate cardiovascular risk, warranting further research across diverse populations.

In our study, ACS was the predominant cardiovascular condition, affecting 39.75% of participants, with no significant gender disparity (40.00% males vs. 39.34% females, p = 0.833). This aligns with findings from several large-scale studies on blood groups and CVDs [6,22,29]. Although we did not find significant associations between ABO blood groups and cardiovascular conditions (p = 0.138), this contrasts with previous research indicating that non-O blood groups generally carry a higher risk of cardiovascular events, with one study attributing approximately 6.27% of coronary heart disease cases to non-O blood group status [29].

Other cardiovascular conditions in our cohort included myocarditis (14.35%), PAD (12.70%), AD (9.35%), CHD (9.10%), tachycardia (8.71%), and arrhythmia (6.04%). While no significant differences were observed across gender or age groups (p > 0.05), some studies report varying risks based on blood type. Non-O blood groups are linked to increased thrombotic events and worse outcomes in heart failure [7,15,30]. Notably, arrhythmia was more prevalent in Rh− individuals (9.29% vs. 5.81%), echoing findings that Rh− status may correlate with poorer ischemic cardiomyopathy outcomes [30].

Our findings regarding ACS showed no significant differences across ABO (p = 0.138) or Rh groups (p = 0.593), which contrasts with studies indicating increased cardiovascular risk in non-O blood groups [7,22]. This discrepancy may be due to population-specific factors or other cardiovascular risk influences [13]. The patterns observed may reflect complex interactions between genetic and environmental factors, as ABO blood group antigens affect physiological processes, including endothelial function and coagulation [6,8,22]. Our study found no significant differences in major cardiovascular conditions across blood groups, but the relationship between ACS and blood type warrants further investigation. These findings highlight the need for more research to explore the underlying mechanisms and improve cardiovascular risk assessment and management.

Limitations

This study has several limitations due to its retrospective design and monocentric nature. These factors may limit the representativeness of the sample and the generalizability of the findings to other hospitals or regions, where genetic and environmental factors may vary. Although the population reflects the diversity of Algiers, caution is needed when extrapolating the results to a national scale. Additionally, our focus on ABO and Rh blood groups may overlook other significant genetic factors influencing cardiovascular risk. The hospitalized patient population may not accurately represent the general population, introducing selection bias that could disrupt HWE and inflate the prevalence of risk factors. Despite these limitations, our study offers valuable insights into the distribution of blood groups among patients with CVDs in Algeria and Africa.

## Conclusions

This study reveals a predominance of blood group O and Rh+ types among hospitalized CVD patients in Algeria; however, no significant associations were identified between blood types and major cardiovascular risk factors such as hypertension, diabetes, dyslipidemia, or obesity. These findings suggest that lifestyle factors and genetic predispositions may have a greater influence on CVD risk. We acknowledge the study's retrospective, monocentric design and potential selection bias, which may limit the generalizability of our findings. For future research, conducting sensitivity analyses could help assess how variations in sample selection might affect the results. Notable observations, such as the high prevalence of hypertension in older males and concerning rates of dyslipidemia and smoking, highlight the urgent need for targeted public health measures. Interestingly, while ACS was the most common condition in this cohort, its lack of association with blood types contrasts with prior studies linking non-O blood groups to increased CVD risk, possibly reflecting unique population characteristics or the multifactorial etiology of CVD. These results underscore the complexity of cardiovascular risk factors and emphasize the importance of actionable recommendations for healthcare professionals, including the promotion of lifestyle interventions and the need for further research to explore the interplay of genetic, environmental, and behavioral influences on CVD.
